# A paediatric patient with PRAKG2 cardiomyopathy: Look at the red flags

**DOI:** 10.1093/ehjcr/ytaf302

**Published:** 2025-06-24

**Authors:** Ana Rita Bello, João Franco-Machado, Inês Carmo Mendes, Bruno M L Rocha

**Affiliations:** Cardiology Department, Hospital de Santa Cruz, ULSLO, Avenida Professor Doutor Reinaldo dos Santos, Carnaxide, 2790-134 Lisbon, Portugal; Clinical Pathology Department, Unidade Local de Saúde de Lisboa Ocidental, Estrada Forte do Alto do Duque, 1449-005 Lisbon, Portugal; Department of Pharmaceutical Sciences and Medicines, Faculty of Pharmacy, Universidade de Lisboa, 1649-003 Lisbon, Portugal; Pediatric Cardiology, Hospital de Santa Cruz, ULSLO, Avenida Professor Doutor Reinaldo dos Santos, Carnaxide, 2790-134 Lisbon, Portugal; Cardiology Department, Hospital de Santa Cruz, ULSLO, Avenida Professor Doutor Reinaldo dos Santos, Carnaxide, 2790-134 Lisbon, Portugal; Cardiomyopathy Outpatient Unit, Hospital de Santa Cruz, ULSLO, Avenida Professor Doutor Reinaldo dos Santos, Carnaxide, 2790-134 Lisbon, Portugal

## Case presentation

A 13-year-old Pakistani boy was referred for cardiovascular screening due to unexplained syncope. The first episodes began at the age of 8 years old, associated with physical activity, and became increasingly more frequent in the last months. He had no other complaints (e.g. fatigue, palpitations, chest discomfort). There was no family history of sudden cardiac death (SCD) nor known structural heart disease.

**Figure 1:**
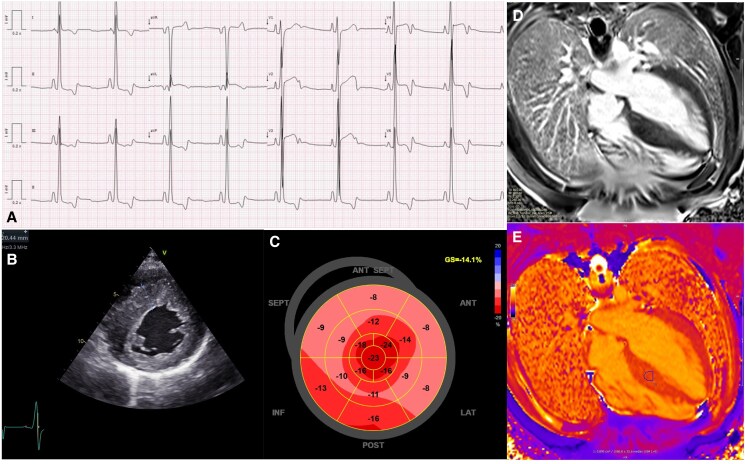
(*A*) ECG—Sinus bradycardia at 50bpm, short PR interval (100 ms), without delta waves (pre-excitation), and difuse biphasic/inversion of T-waves (pre-cordial leads, DI and aVL leads and V_4-6_ and DII), and borderline QTc (automatic 452 ms). (*B*) TTE—short axis demonstrating increased asymmetrical septal LV wall thickness (19 mm; *z* score 6). (*C*) TTE—reduced global longitudinal strain (−14.1%). (*D*, *E*) Cardiac magnetic resonance—presence of mild patchy late-gadolinium enhancement in the interventicular septum and increased T1 mapping values (*E*, circle) in the same region; there were no morphological signs suggestive of sarcomeric hypertophic cardiomyopathy; global T1 mapping values were within normal range (1010 ms; reference centre: 900 to 1050 ms).

The ECG (*Figure 1A*) showed sinus bradycardia, short PR interval, no pre-excitation, and biphasic T-waves. The transthoracic echocardiogram showed severe left ventricular (LV) hypertrophy, preserved LV ejection fraction, and mildly reduced global longitudinal strain (*Figure 1B*; Supplementary Material online, *Video S1A–C*). On magnetic resonance, there was discrete late gadolinium enhancement in the septum, with normal global T1 mapping values (*Figure 1D* and *E*). The laboratory assessment showed a slightly elevated NT-proBNP (230 pg/mL) and spurious proteinuria (urine protein-to-creatinine ratio: 2105 mg/g). The latter, most probably, due to analytical interference of a glycogen metabolite (see Supplementary material online, *Figure S1*).

Following genetic counselling, the patient underwent testing following a hypertrophic cardiomyopathy panel including 38 genes (see Supplementary Material online), revealing a heterozygous pathogenic variant in the PRKAG2 gene [OMIM#602743] [NM_016203.4:c.905G > A (p.Arg302Glu)]. His parents tested negative for this variant, supporting a *de novo* mutation. Recurrent syncope on exertion, massive LV hypertrophy and PRKAG2 Glycogen Storage Disease Cardiomyopathy were considered to potentially increase the risk of SCD, despite no malignant arrhythmias on 48h-Holter monitoring and exercise stress test. Accordingly, the multidisciplinary discussion favoured the use of an implantable cardio-defibrillator. A transvenous approach was preferred, since SCD is reported in up to 10% of the patients, often due to advanced heart block^1,2^

This case underscores the importance of the genetic aetiological identification in paediatric cardiomyopathy. Moreover, it suggests that spurious proteinuria, arising from the interference of a glycogen metabolite, may be a potential new diagnostic ‘red flag’.^3^

## Supplementary Material

ytaf302_Supplementary_Data

## Data Availability

The data underlying this article are available in the article and in its online *Supplementary material*.

## Notes

**Consent:** The patient and his legal tutor verbally consented to the publication of his medical case in a peer-reviewed Medical Journal. The authors of this article, who actively participated in the decision process and management, obtained the written informed consent from the patient, in accordance with COPE guidelines.

**Funding:** None declared.
