# Supplementary material for: A paediatric patient with PRAKG2 cardiomyopathy: Look at the red flags
Source: Eur Heart J Case Rep. 2025 Jun 24;9(7):ytaf302. doi: 10.1093/ehjcr/ytaf302 (PMC12244331; doi:10.1093/ehjcr/ytaf302)
Supplement: ytaf302_Supplementary_Data [file ytaf302_supplementary_data.zip › SUPPLEMENTARY MATERIAL.docx]

**[SUPPLEMENTARY MATERIAL]:**

**[SUPPLEMENTARY FIGURE LEGEND]:** Red flags for PRKAG2 cardiomyopathy.

**Identification of spurious proteinuria**: Proteinuria was initially assessed by imunoturbidimetry, using benzethonium chloride in alkaline medium (450 mg/dL; urine protein-to-creatinine ratio: 2105 mg/g). Parallelly, urinalysis (semi-quantitative tetrabromophenol blue strip) showed an incongruent result, with vestigial proteinuria (15 to 25 mg/dL), which led us to further investigate through alternative analytical methods. Biuret method (copper sulfate in alkaline medium, in the presence of potassium iodate and sodium potassium tartrate) also showed high proteinuria (1160 mg/dL; urine protein-to-creatinine ratio: 5420 mg/g), but urine proteins were found within the normal range on capillary electrophoresis (18 mg/dL; urine protein-to-creatinine ratio: 84 mg/g) and on colorimetry using pyrogallol red and sodium molybdate (29 mg/dL; urine protein-to-creatinine ratio: 135 mg/g). These findings raised suspicions on spurious results obtained from both the immunoturbidimetric and Biuret methods. Indeed, there is known interference in the Biuret method caused by reducing carbohydrates, such as glycogen and its metabolites. We suspected the presence of glucose tetrasacharide (Glc_4_) in the urine, as was previously reported in other glycogenosis (e.g.: Pompe disease, Cori-Forbes disease).^3^ We presume this glycogen metabolite as the most probable cause of the observed analytical interference, after excluding other possible causes (such as known drugs or medical procedures), although mass spectrometry was not conclusive.

**Hypertrophic Cardiomyopathy Gene Panel used (38 genes)**: ACTC1, ACTN2, ALPK3, ANKRD1, CACNA1C, CALR3, CAV3, CSRP3, FHL1, FHOD3, FLNC, GLA, JPH2, KLHL24, LAMP2, LDB3, MYBPC3, MYH6, MYH7, MYL2, MYL3, MYLK2, MYOZ2, MYPN, NEXN, PDLIM3, PLN, PRKAG2, RYR2, TCAP, TNNC1, TNNI3, TNNT2, TPM1, TRIM63, TTN, TTR, VCL.

**[SUPPLEMENTARY VIDEOS LEGEND]:**

**Video 1**. Transthoracic Echocardiogram: **A** – Parasternal long axis (PLAX) view; **B** – 4-chamber (4C) view; **C** – 2-chamber (2C) view; severe LV hypertrophy (interventricular septum = 19mm, z-score 6, indexed LV mass 138g/m2). Preserved LV ejection fraction (Simpson biplane 63%). E/A 2.2; E/e’ = 9. RV with preserved longitudinal function.
