# Supplementary figures and images for: A paediatric patient with PRAKG2 cardiomyopathy: Look at the red flags
Source: Eur Heart J Case Rep. 2025 Jun 24;9(7):ytaf302. doi: 10.1093/ehjcr/ytaf302 (PMC12244331; doi:10.1093/ehjcr/ytaf302)

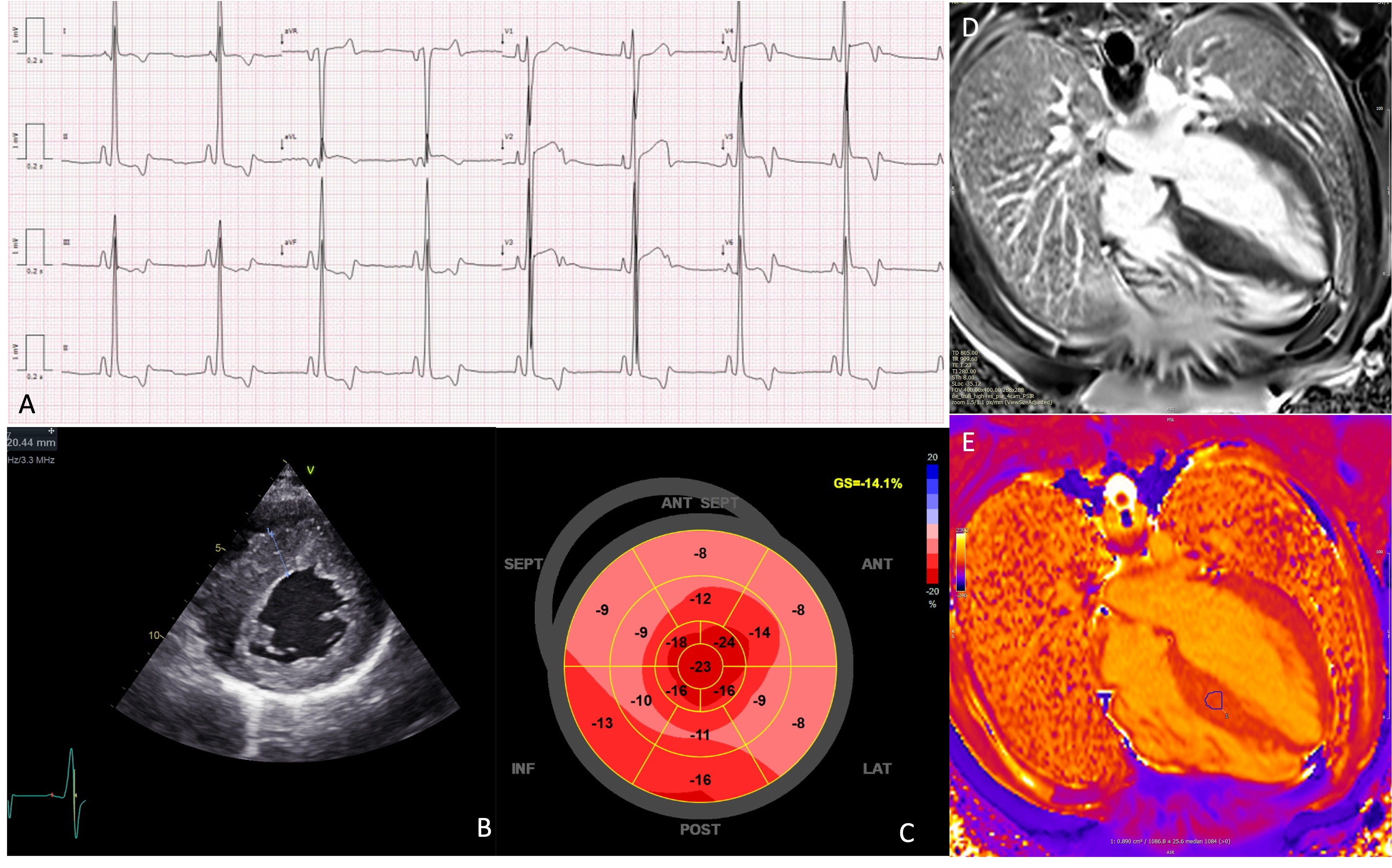

Supplement: ytaf302_Supplementary_Data [file ytaf302_supplementary_data.zip › Image1.tiff]

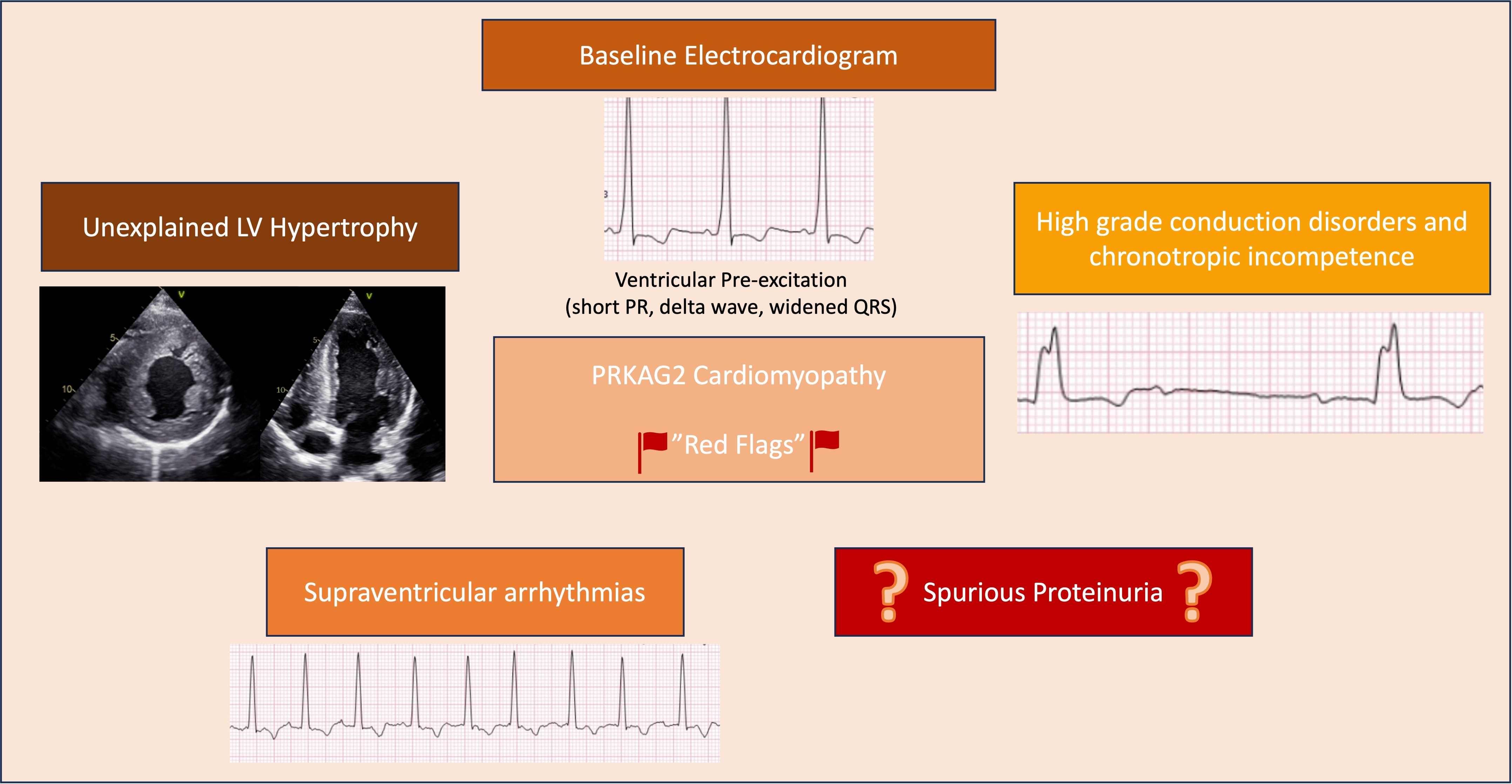

Supplement: ytaf302_Supplementary_Data [file ytaf302_supplementary_data.zip › SupFigure.tiff]
